# Our New York Letter

**Published:** 1890-07

**Authors:** 


### Correspondence.

### OUR NEW YORK LETTER.

NEW YORK, June 16, 1890.

Dr. H. Holbrook Curtis, the eminent laryngologist, of this city, who, by the way, has demonstrated his originality of thought and fertility of invention in another and different department of medicine by being the author of the method of deep irrigation of the urethra with bichloride solution for the treatment of acute gonorrhœa, and which has been recently highly commended by Professor Otis in a clinical lecture, has recently given expression to his views on the modern treatment of pneumonia. With the large practical experience of Dr. Curtis in the treatment of this affection, these views must naturally commend themselves to the practical physician, founded as they are on well established data and supported by clinical study and observation.

Dr. Curtis deprecates the use of a large majority of the numerous proprietary hypnotics and antipyretics at present employed by the profession by reason of their tendency to cardiac ennervation. He relies wholly on the use of digitaline, strychnine and aconitine in the treatment of pneumonia, for in their administration, he states, we have the heart-stimulant par excellence. In patients who have been addicted to the excessive use of alcohol previous to an attack of pneumonia, the combination of digitaline and strychnine gives the best results, overstimulation from digitaline being quickly corrected by the free administration of aconitine, which dilates the peripheral arterioles and equalizes the circulation. He prefers the use of aconitine and digitaline to the tincture of aconite or digitalis.

He further says that aconitine, when administered in small and repeated doses during the febrile state, exerts its force in combatting increased tissue metamorphosis, and thus reduces the pyrexia.

It has been Dr. Curtis' custom for the past three years, in the treatment of acute pneumonia, to give a dose of aconitine sufficient to reduce the temperature to 100 deg. F., commencing with 1-130 gr. (Chanteaud), administered either hourly or halfhourly, but always in combination with 1-64 gr. of digitaline and 1-64 gr. of arsenate of strychnine. He gives the alkaloids at indicated intervals until the temperature falls to 100 deg F., at which time the aconitine is to be suspended, and when the pulse falls to 90 deg., the digitaline is to be dropped, the strychnine, however, to be continued, at hourly intervals, throughout the attack.

In the case of the aged especially, Dr. Curtis recommends his method of treating pneumonia, and states that no careful observer who has ever used these drugs in combination and noted their action on the heart, will ever be inclined to place much confidence in alcohol and ammonia in the treatment of this affection.

He cites one great advantage in using the active principle in these drugs, which is that the stomach is hardly ever disturbed thereby, the granules being so minute that they may be taken when there is no toleration of either nourishment or fluids of any description. The objection made to the use of these alkaloids on account of their supposed causation of nephritis, he regards as wholly untenable, for after a very large experience in their administration and careful attention given in the analysis of the urine in every case in which they have been heroically pushed, he found no evidence to justify such an allegation.

Dr. Thomas H. Manley, visiting surgeon to the Harlem Hospital, speaking at a recent clinic in reference to cocaine anæsthesia, said that his attention had been particularly directed to the anelgesic action of this drug by a very interesting article from the pen of Prof. Paul Rechus, of the Hotel Dieu, Paris, which appeared in a recent issue of the *Mercerdi Medicale.* The writer had reported the results of 700 consecutive operations in which cocaine had been employed as the anæsthetic. Dr. Manley said that he himself had operated with gratifying success a short time since on a patient for the radical cure of hernia, using cocaine as an anæsthetic for that purpose. The patient was a young man who had an incarcerated direct inguinal hernia, but on account of an injury to his skull, which he sustained some years previously, he was desirous of dispensing with ether or chloroform. Cocaine was accordingly employed, and one drachm of a five per cent. solution of the drug was injected by means of a long needle, after the plan recommended by Rechus, covering the entire area of the part to be operated upon. The needle was plunged deeply into the tissues, and, as the piston was compressed, the needle was gradually withdrawn, so that the whole line of puncture was effectually saturated with the solution. After waiting about fifteen minutes, the cutting operation was begun. The hernial sac was found to be an old one, in which the spermatic cord was intimately associated by old adhesions, and a very tedious and careful dissection was necessary in order to separate it and free the sac previous to excision and ligation. The operation occupied about three-quarters of an hour, and during the entire period the patient presented no evidence whatever of pain or discomfort.

Dr. Manley referred to the great advantage cocaine possesses over either of the other two anæsthetics in a country village or town practice, where a physician is sometimes called upon to do an operation in an emergency, and where skilled or unskilled attendants are difficult or impossible to obtain. In the use of cocoaine he has an agent by the use of which he can readily paralyze the part over which he proposes to operate, and he has, besides, the additional advantage of the intelligent co-operation of the patient himself.

Dr. Manley also performed a few days ago, a laparotomy for the evulsion of an ovarian cyst by the use of a three per cent. solution of cocoaine, and with the most gratifying results. He maintains that in the performance of laparotomy for exploratory purposes alone, cocoaine will prove satisfactory in every instance; but where the operation is tedious, extending over an hour or more, he then advises the use of chloroform or ether.

The constitutional effects of the drug on those patients operated upon by him were in no instance very marked. The only condition he noticed was a decided local paralysis of the reflexes in the field of operation.

Dr. Gleitzaman, at a recent meeting of the section of laryngology at the Acadamy of Medicine, called the attention of the members present to a new remedy for the removal of hypertrophical growths in the nasal cavity that he had been using with very gratifying results during the past six weeks. It is known by the name of trichloro-acetic acid. He has employed it so far in 98 patients, and in but two of these did the resulting scar liquify and give rise to catarrhal symptoms. As a rule, the eschar remains dry; the patient is not aware that any application has been made to the parts; no catarrhal symptoms follow, and in six or seven days after the application of the acid to the nasal cavity, the scar can be removed with a pair of forceps.

The following liniment has been found very effective for the treatment of acute synovitis, and is largely employed here in hospital practice. Sponges soaked in the solution are placed around the affected joint, and over these is carried a firmly applied roller bandage:

℞ Lin. belladonna,<br>

Lin. aconiti, } aa. ℥ ij.<br>

Glycerini,<br>

M. S.

In twenty-four to forty-eight hours after its use the pain and swelling will be found to have entirely disappeared, and the patient will experience a considerable amount of relief in consequence. J. P. R.

---

### A VALUABLE REMEDY FOR BRAIN-WORKERS.

---

We have received several inquiries concerning a much advertised secret remedy which has found great favor among the clergy in the West, possibly because they have a percentage on all the custom which they can bring to the "professor." The remedy is sent, on the receipt of a pledge of secrecy, to anyone for the trifling sum of $10, $6 of which is given to the clergyman who secures the order. Sometimes it is the clergyman himself who is sold. A correspondent of the *Medical Standard* says that the remedy is nothing more or less than a daily enema of two gallons of water, "persistence in which," the accompanying pamphlet says, will remove the microbes of the intestinal tract from the stomach down."—*Medical Record.*

